# Excess Mortality in Kidney and Kidney-Pancreas Transplant Recipients in the COVID-19 Pandemic in Portugal—A Cohort Study

**DOI:** 10.3389/ti.2023.11655

**Published:** 2023-09-29

**Authors:** Miguel T. Coimbra, José A. T. S. Francisco, Joana C. Freitas, Renata V. Carvalho, Sara R. B. Vilela, Catarina I. C. D. Ribeiro, José L. C. S. L. Silvano, Sofia Pedroso, Manuela Almeida, La Salete Martins, Jorge Malheiro

**Affiliations:** ^1^ Department of Nephrology, Centro Hospitalar Universitário de Santo António, Porto, Portugal; ^2^ Department of Nephrology, Hospital Espírito Santo de Évora, Évora, Portugal; ^3^ Department of Nephrology, Centro Hospitalar de Trás-os-Montes e Alto Douro, Vila Real, Portugal; ^4^ Department of Nephrology, Hospital de Braga, Braga, Portugal; ^5^ Department of Nephrology, Hospital Garcia de Orta, Almada, Portugal

**Keywords:** excess mortality, kidney transplant, COVID-19, mortality, pancreas allograft

## Abstract

The COVID-19 pandemic increased morbidity and mortality worldwide, particularly in the Kidney and Kidney-Pancreas Transplant Recipient (KTR/KPTR) population. Aiming at assessing the absolute and relative excess mortality (EM) in a Portuguese KTR/KPTR cohort, we conducted a retrospective observational study of two KTR/KPTRs cohorts: cohort 1 (*P1*; *n* = 2,179) between September/2012 and March/2020; cohort 2 (*P2*; *n* = 2067) between March/2020, and August/2022. A correlation between relative and absolute EM and age, sex, time from transplantation and cause of death was explored. A total of 145 and 84 deaths by all causes were observed in *P1* and *P2*, respectively. The absolute EM in *P2* versus *P1* was 19.2 deaths (observed/expected mortality ratio 1.30, *p* = 0.006), and the relative EM was 1.47/1,000 person-months (95% CI 1.11–1.93, *p* = 0.006). Compared to the same period in the general population, the standardized mortality rate by age in *P2* was 3.86 (95% CI 2.40–5.31), with a peak at 9.00 (95% CI 4.84–13.16) in *P2*C. The higher EM identified in this population was associated, mainly, with COVID-19 infection, with much higher values during the second seasonal COVID-19 peak when compared to the general population, despite generalized vaccination. These highlight the need for further preventive measures and improved therapies in these patients.

## Graphical Abstract

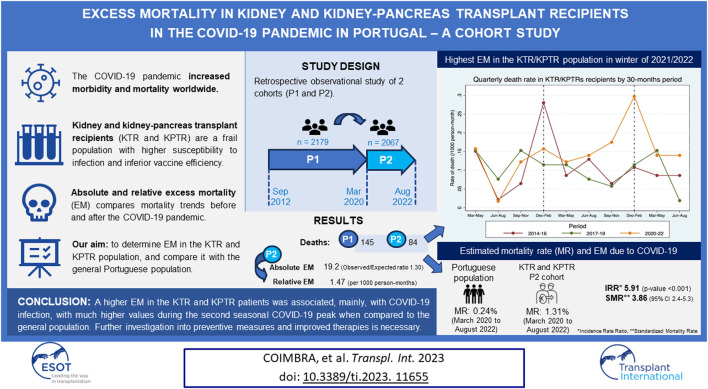

## Introduction

Severe acute respiratory syndrome coronavirus 2 (SARS-CoV-2) is the highly contagious etiological agent of the novel coronavirus disease 2019 (COVID-19), which was responsible for the unprecedented pandemic that started at the beginning of 2020.

In Portugal, epidemiologic studies in the general population reported a total of 5,417,101 confirmed cases and 24,855 deaths due to COVID-19 between March 2020, and August 2022 [1]. In 2021, the number of deaths attributed to COVID-19 noticeably increased to 12,004 (9.6% of all deaths), when compared to a total of 6,972 deaths reported due to COVID-19 in 2020, and January 2021 was the month with the highest number of deaths due to COVID-19 (*n* = 5,804) [1, 2]. We noticed that, in the second winter, January 2022 registered the highest monthly record of infections (1,277,754 confirmed cases) but only 1,002 deaths, representing an estimated 82.7% reduction in COVID-19-related deaths compared to the homologous month of the previous year. The emergence of vaccination strategies against SARS-CoV-2 and less lethal SARS-COV-2 variants were believed to have significantly reduced the morbidity and mortality associated with COVID-19. As countries worldwide started waiving the tight sanitary measures and progressively lifting the protective measures in place in 2022, it has led to increased exposure of SOT recipients, who are immunosuppressed patients prone to infection in general and to SARS-CoV-2 in particular. The morbidity and mortality associated with COVID-19 have been the subject of great concern in kidney transplant and kidney-pancreas transplant recipients (KTR/KPTRs), and we believe it is crucial to assess the impact of COVID-19 infection in this frail patient population, whether mortality and EM during the COVID-19 pandemic in these patients differed from the general public, and to discuss the role of preventive measures such as vaccination in KTRs.

This study aims to investigate the mortality rate and EM in the KTR/KPTR patient population during the COVID-19 pandemic and compare them with those of the general Portuguese population.

## Materials and Methods

This was a retrospective cohort study of patients who received a kidney or kidney and pancreas transplant before August 2022, at Centro Hospitalar Universitário de Santo António (CHUSA) and remained active transplant recipients between September 2012, and August 2022. Patients with time since last transplant less than 6 months were excluded. Data were retrieved from the Portuguese Transplantation Registry (*Registo Português de Transplantação*, RPT), Portuguese Electronic Health Registry (*Registo de Saúde Eletrónico,* RSE), and CHUSA database.

Two patient cohorts were considered pertaining to two time intervals: one comprising 90 months before the COVID-19 pandemic (i.e., between September 2012, and March 2020; pre-pandemic cohort [P1]), and another comprising 30 months of COVID-19 pandemic (i.e., between March 2020, and August 2022; pandemic cohort [P2]).

A time-dependent prospective model was used to calculate expected deaths in the kidney transplant population between cohorts. We first determined the likelihood of death in person-months among a database of observed deaths and mortality rate in a given time-frame (between September 2012 and March 2020, among kidney transplant recipients with time of transplant prior to March 2020), adjusted by exposure (months since time of transplant) during the pre-pandemic cohort (P1). Our survival model allowed us to estimate the expected absolute mortality and the absolute observed/expected mortality (O/E) ratio in the COVID-19 era, using a second dataset of observed deaths (both COVID-19 and non-COVID-19 related deaths). By establishing the mortality rate among KTR included in the second cohort (P2), absolute EM was estimated by subtracting the expected absolute mortality from the observed absolute mortality. We were able to determine the relative excess mortality (or incidence rate ratio, *IRR)* in the KTR population during the COVID-19 pandemic (P2), after adjusting observed deaths (between March 2020 and August 2022) to exposure (months since time of transplant), and comparing both mortality rates in person-months (incidence rates, *IR*) between our two different time-frames (P1 and P2). The mortality rate per 1,000 person-months and incidence rate ratio (IRR) in P1 and P2 were estimated with a 95% confidence interval. We used a univariate analysis to adjust our EM findings to several variables, including age (<20 years, 20–40 years, 40–50 years, 50–60 years, 60–70 years, >70 years), sex, time from transplantation (0–6 months, 6–12 months, 12–24 months, 24–48 months, 48–120 months, >120 months), and cause of death (cardiovascular disease, malignancy, COVID-19 infection, non-COVID-19 infection, all infections, other causes).

EM in the KTR population during the pandemic (P2) was further assessed according to three consecutive 10-month intervals: P2A (from March to December 2020), P2B (from January to October 2021), and P2C (from November 2021 to August 2022).

The significance as a function of the considered variables in EM was estimated using the Chi-square test (x^2^).

Statistical analysis was performed using Stata® for Windows.

Publicly available sources (Statistics Portugal [*Instituto Nacional de Estatística*, INE], the Directorate-General of Health [*Direção Geral de Saúde*, DGS], and other readily available online sources) were used to retrieve epidemiological data regarding incidence peaks of COVID-19 infection and COVID-19 vaccination rates and coverage in the general Portuguese population. The EM identified in the KTR study cohort between March 2020, and August 2022, was compared to EM of the general Portuguese population.

## Results

A total of 2,179 KTR/KPTRs (corresponding to an exposure of 144.641 person-months) were included in P1, and 2,067 KTR/KPTRs (corresponding to an exposure of 57.080 person-months) were included in P2.

### Global Excess Mortality in the KTR/KPTR Population Before (P1) and During (P2) COVID-19 Pandemic

The absolute and relative EM found in the KTR/KPTR population *in P1 and P2 is* depicted in Tables 1, 2, respectively.

**TABLE 1:** Absolute excess mortality in the KTR/KPTR population in the P2 period.

Subgroup	*P1*	*P2*				
	Observed (deaths)	Observed (deaths)	Expected (deaths)	Excess	O/E death ratio	*p*-value
Total	145	84	64.8	19.2	1.30	0.006
*P2A*	—	21	18.9	2.1	1.11	0.621
*P2B*	—	26	19.8	6.2	1.31	0.154
*P2C*	—	37	21.6	15.4	1.71	0.001
Age						
0–20	0	1	0.3	0.7	3.33	0.271
20–40	12	4	4.3	−0.3	0.93	0.558
40–50	17	9	6.7	2.3	1.34	0.311
50–60	29	13	12.2	0.8	1.07	0.789
60–70	38	29	20	9.0	1.45	0.020
70+	49	28	22.8	5.2	1.23	0.204
Sex						
Male	84	50	37.9	12.1	1.32	0.024
Female	61	34	26.9	7.1	1.26	0.113
Time since last KT						
0–6 months	11	8	4.3	3.7	1.86	0.048
6–12 months	4	1	1.1	−0.1	0.91	0.694
12–24 months	5	1	1.3	−0.3	0.77	0.626
24–48 months	12	7	4.0	3.0	1.75	0.084
48–120 months	44	21	14.7	6.3	1.43	0.047
+120 months	69	46	31.1	14.9	1.48	0.003
Cause of death						
Global	145	84	64.8	19.2	1.30	0.006
Cardiovascular	45	18	17.8	0.2	1.01	0.961
Malignancy	34	12	13	−1.0	0.92	0.737
COVID-19 infection	36	27	17.8	9.2	1.52	0.014
No COVID-19 infection	36	13	13.9	−0.9	0.94	0.782
All infections	36	40	21.5	18.5	1.86	<0.001
Other causes	30	14	12.5	1.5	1.12	0.608

KPTR, Kidney and pancreas transplant recipient; KT, Kidney transplant; KTR/KPTRs, Kidney/kidney-pancreas transplant recipients; O/E, observed/expected; P1, Pre-COVID-19 pandemic period; P2, COVID-19 pandemic period; P2A, March to December 2020 pandemic period; P2B, January to October 2021 pandemic period; P2C, November 2021 to August 2022 pandemic period.

**TABLE 2:** Mortality rates in the KTR/KPTR population in P1 and P2 periods, and relative excess mortality in P2.

Subgroup	*P1*			*P2*			IRR (95% CI)	*p*-value
	Observed (deaths)	Exposure (person-months)	IR (per 1,000 person-months)	Observed (deaths)	Exposure (person-months)	IR (per 1,000 person-months)		
Total	145	144,641	1.003	84	57,080	1.472	1.468 (1.108–1.934)	0.006
*P2A*	—	—	—	21	18,631	1.127	1.124 (0.675–1.784)	0.602
*P2B*	—	—	—	26	18,929	1.374	1.370 (0.866–2.090)	0.149
*P2C*	—	—	—	37	19,459	1.901	1.897 (1.285–2.738)	0.001
Age								
0–20	0	2,190	0	1	814	1.229	NA	0.271
20–40	12	18,357	0.654	4	6,781	0.590	0.902 (0.212–2.977)	0.895
40–50	17	32,816	0.518	9	11,319	0.795	1.535 (0.603–3.640)	0.307
50–60	29	36,478	0.795	13	14,944	0.870	1.094 (0.522–2.172)	0.774
60–70	38	32,616	1.165	29	13,887	2.088	1.792 (1.066–2.984)	0.021
70+	49	2,285	2.209	28	9,335	3.000	1.358 (0.822–2.204)	0.201
Sex								
Male	84	88,262	0.952	50	34,829	1.436	1.508 (1.041–2.166)	0.024
Female	61	56,288	1.084	34	22,209	1.531	1.413 (0.900–2.183)	0.112
Time since last KT								
0–6 months	11	4,721	2.330	8	1,394	5.739	2.463 (0.860–6.723)	0.064
6–12 months	4	9,128	0.438	1	2,589	0.386	0.881 (0.018–8.907)	0.981
12–24 months	5	17,871	0.280	1	4,808	0.208	0.743 (0.016–6.643)	0.865
24–48 months	12	34,235	0.351	7	9,086	0.770	2.198 (0.733–6.055)	0.114
48–120 months	44	71,204	0.618	21	20,799	1.010	1.634 (0.923–2.807)	0.072
+120 months	69	86,893	0.794	46	32,182	1.429	1.800 (1.212–2.652)	0.003

CI, Confidence interval; IR, Incidence rate; IRR, Incidence rate ratio; KTR/KPTRs, Kidney/kidney-pancreas transplant recipients; KT, Kidney transplant; KTR, Kidney transplant recipient; NA, Not attributed; P1, Pre-COVID-19 pandemic period; P2, COVID-19 pandemic period; P2A, March to December 2020 pandemic period; P2B, January to October 2021 pandemic period; P2C, November 2021 to August 2022 pandemic period.

Overall, 145 and 84 deaths by all causes were observed in this patient population in P1 and P2, respectively (Tables 1 and 2). The absolute EM in P2 compared to P1 was 19.2 deaths (O/E ratio 1.30, *p* = 0.006), and the relative EM was 1.47 per 1,000 person-months (95% CI 1.11–1.93, *p* = 0.006).

Stratifying the analysis according to the three pandemic time intervals considered, 21, 26, and 37 deaths by all causes were identified in P2A, P2B, and P2C, respectively (Tables 1, 2). P2C had the highest absolute and relative EM of the three time periods. Compared to P1, the absolute EM was 2.1 deaths in P2A (O/E ratio, 1.11, *p* = 0.621), 6.2 deaths in P2B (ratio O/E, 1.32, *p* = 0.154), and 15.4 deaths in P2C (O/E ratio, 1.71, *p* = 0.001). This translates into a relative EM of 1.12 in the pandemic period P2A (*p* = 0.602), 1.37 in P2B (*p* = 0.149), and 1.90 in P2C (*p* = 0.001) compared to before the pandemic (P1).

**FIGURE 1:**
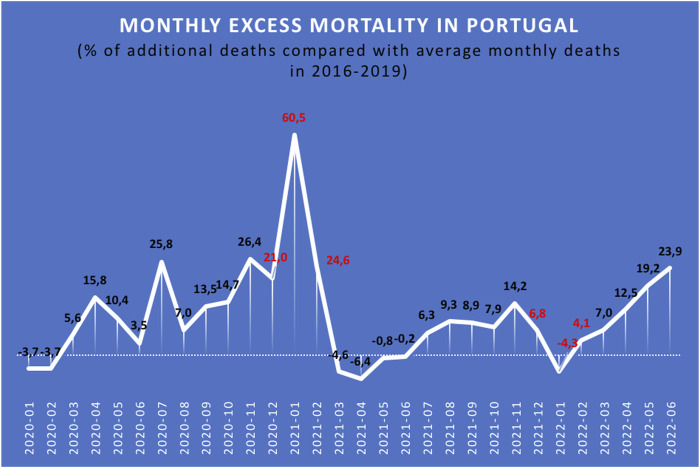
Relative excess mortality (%) in the Portuguese population during the COVID-19 pandemic [3].

The highest EM in the KTR/KPTR population during the COVID-19 pandemic was seen in 2021/2022 winter, with a mortality rate approximately 3 times higher than that of the two previous pre-pandemic periods (Figure 1). Conversely, in 2020/2021 winter, only a moderate mortality increase was observed in this patient population [3]. An opposite trend was observed in the general Portuguese population during the pandemic, with the highest EM recorded in 2020/2021 winter (20–60% EM compared to the previous winter), and only a marginal increase in EM (−4 to +6%) seen in 2021/2022 winter compared to the last pre-COVID-19 winter.

### Excess Mortality by Age of Transplant Recipients

An absolute EM (i.e., >0 deaths and/or O/E ratio >1.0) was identified in most age subgroups assessed. The exception was the 20–40 year-old subgroup, where the O/E ratio was 0.93. However, statistical significance was only achieved in the subgroup of patients aged between 60–70 years, where an observed absolute mortality of 29 deaths, an expected absolute mortality of 20 deaths, and an O/E ratio of 1.45 (*p* < 0.020) were reported, together with an increase in relative EM (IRR 1.792, 95% CI, 1.066–2.984, *p* = 0.021) (Tables 1, 2).

### Excess Mortality by Sex of Transplant Recipients

An absolute EM was observed in both female and male transplant recipients, with a higher number of observed and expected deaths in men (50 and +37.9, respectively) compared to women (34 and +26.9, respectively). IRR was also higher in men (1.508, 95% CI 1.041–2.166 vs. 1.413; 95% CI 0.900–2.183 in women), but the mortality rate per 1,000 person-months was lower (IR 1.436 vs. 1.531 in women). However, this difference was not statistically significant (*p* = 0.112) (Tables 1, 2).

### Excess Mortality by Time Since the Last Active Kidney Transplant

In this study, KTRs who received an active transplant less than 6 months before August 2022 had a significant increase in absolute EM (O/E ratio 1.86, *p* = 0.048). KTRs with an active transplant for 48–120 months and >120 months also showed a significant rise in the number of deaths and absolute EM (O/E ratio 1.43, *p* = 0.047 and O/E ratio 1.48, *p* = 0.003, respectively). The mortality rate in KTRs with >120 months of active kidney transplant was 1.429 per 1,000 person-months, and the relative EM was highest in this patient subgroup (IRR 1.800, 95% CI 1.212–2.652, *p* = 0.003). Interestingly, there was no absolute or relative EM in the periods between 6 and 12 months of active kidney transplant (O/E ratio 0.91, *p* = 0.694; IRR 0.881, *p* = 0.981) or between 12 and 24 months of active transplant (O/E ratio 0.77, *p* = 0.626; IRR 0.743, *p* = 0.865) (Tables 1, 2).

### Excess Mortality by Non-COVID-19 Causes

The absolute and relative EM due to cardiovascular causes, malignancy, or other non-COVID-19 causes was not statistically significant (*p* > 0.5 for all subgroups). In the subgroup of death by all causes of infection, the absolute EM was +18.5 (O/E ratio 1.86, *p* < 0.001), and IRR was 2.82 (95% CI 1.750–4.546, *p* < 0.001). However, when excluding deaths attributed to COVID-19 infection in *P2*, the absolute and relative EM due to all causes of infection (excluding COVID-19) was not statistically significant (*p* > 0.5) (Tables 1, 3)

**TABLE 3:** Relative excess mortality in the KTR/KPTR population in P1 and P2 periods according to cause of death.

Subgroup	*P1*		*P2*		IRR (95% CI)	*p*-value
	Observed (deaths)	IR (per 1,000 person-months)	Observed (deaths)	IR (per 1,000 person-months)		
Cause of death						
Global	145	1.003	84	1.472	1.468 (1.108–1.934)	0.006
Cardiovascular	45	0.311	18	0.315	1.014 (0.552–1.786)	0.946
Malignancy	34	0.235	12	0.210	0.894 (0.422–1.771)	0.759
COVID-19 infection	NA	NA	27	0.473	1.901 (1.110–3.219)	0.014
No COVID-19 infection	36	0.249	13	0.228	0.915 (0.445–1.767)	0.802
All infections	36	0.249	40	0.701	2.816 (1.750–4.546)	<0.001
Other causes	30	0.207	14	0.245	1.183 (0.579–2.300)	0.597

CI, Confidence interval; IR, Incidence rate; IRR, Incidence rate ratio; KTR/KPTRs, Kidney/kidney-pancreas transplant recipients; NA, Not attributed; P1, Pre-COVID-19 pandemic period; P2, COVID-19 pandemic period.

### Excess Mortality by COVID-19 Infection

Of the total 84 deaths observed in P2, 32% (*n* = 27) were due to COVID-19 infection. The analysis by 10 months periods showed that 5 out of 21 deaths (24%) in P2A, 4 out of 26 deaths (15%) in P2B, and 18 out of 37 deaths (49%) in P2C were attributed to COVID-19. Among deaths due to infection (*n* = 40), nearly 68% (*n* = 27) were attributed to COVID-19 infection (Tables 1, 3, 4).

**TABLE 4:** Mortality due to COVID-19 infection in KTR/KPTRs and in the general Portuguese population between March 2020 and August 2022.

Subgroup	General Portuguese population			KTR/KPTR population				IRR	*p*-value	SMR by age (95% CI)
	Observed (deaths)	Mortality rate (%)	Subgroup	Observed (deaths)	Mortality rate (%)	Expected (deaths)	Excess			
T	24,855	0.24	T	27	1.31	4.6	22.4	5.91	<0.001	3.86 (2.40–5.31)
A	6,906	0.07	*P2A*	4	0.21	1.2	2.8	3.22	0.050	2.50 (0.31–4.69)
B	11,250	0.11	*P2B*	5	0.26	2.0	3.0	1.96	0.074	1.33 (0.03–2.64)
C	6,699	0.07	*P2C*	18	0.91	1.3	16.7	14.19	<0.001	9.00 (4.84–13.16)

A, COVID-19 period between March 2020 and December 2020; B, COVID-19 period between January 2021 and October 2021; C, COVID-19 period between November 2021 and August 2022; IRR, Incidence rate ratio; KTR/KPTRs, Kidney/kidney-pancreas transplant recipients; P2A, March to December 2020 pandemic period; P2B, January to October 2021 pandemic period; P2C, November 2021 to August 2022 pandemic period; SMR, Standardized mortality rate; T, COVID-19 period between March 2020 and August 2022.

In a stratified correlation analysis between EM and cause of death, the expected deaths by COVID-19 infection in transplant recipients reached 17.8 (vs. 27 observed deaths), with an O/E ratio of 1.52 (*p* = 0.014). The mortality rate due to COVID-19 in transplant recipients was 0.473 per 1,000 person-months, and the IRR was 1.901 (95% CI 1.110–3.219).

According to data from INE, DGS, and other online sources [4], 24,855 deaths due to COVID-19 infection were recorded in the general Portuguese population (10,206,016 inhabitants) between March 2020 and August 2022, accounting for a mortality rate of approximately 0.24% (Table 4). In a subanalysis, the observed deaths and mortality rate due to COVID-19 infection in the KTR population in P2 were directly compared with those of the general Portuguese population, considering the same 10-month stratification periods: period A, between March 2020, and December 2020; period B, between January 2021, and October 2021; and period C, between November 2021, and August 2022; Table 4), and the pre-COVID cohort (P1) was not included in this comparison. Overall, relative EM was estimated with an IRR of 5.91 due to COVID-19 infection between March 2020 and August 2022 and a standardized mortality rate (SMR) by age of 3.86 (95% CI 2.40–5.31, *p* < 0.001). A much higher EM was observed during period C (+16.7) compared to periods A and B (+2.8 and +3.0, respectively) in the KTR/KPTR population, and a relatively high EM due to COVID-19 was also observed during period C in transplant recipients compared to the general population (IRR 14.19, SMR 9.00, 95% CI 4.84–13.16, *p* < 0.001).

## Discussion

The emergence of vaccination strategies against SARS-CoV-2 significantly reduced the morbidity and mortality associated with COVID-19 in the general public. A large-scale observational US study from 2021 reported a mortality reduction of over 80% in SARS-CoV-2-vaccinated individuals (i.e., with two doses of the mRNA Pfizer-BioNTech® vaccine, two doses of the mRNA Moderna^®^ vaccine, or one dose of the Johnson & Johnson^®^ adenovirus vaccine) [5]. In Portugal, the vaccination campaign started on December 2020 [6]. In our experience, transplant recipients were among the first citizens eligible to receive their first dose of vaccination, as they are a priority group, and started receiving the boost dose by October 2021. By December 2021, nearly all patients had at least full dose vaccination for COVID-19.

According to several sources [3, 7], the monthly excess mortality (EM) associated with the COVID-19 pandemic in Portugal was much higher in January 2021 (between 21.0% and 60.5%) than in January 2022 (between −4.3% and 6.8%) (Figure 1) [3]. Possible explanations for this EM reduction are vaccine effectiveness in the general Portuguese population, on the one hand, and the emergence and implementation of the SARS-CoV-2 Omicron variant, on the other, as a fast-spreading variant that several authors argue is less lethal than its Delta variant predecessors [8, 9].

In our KTR cohort in the pandemic period and until August 2022, we found an absolute EM of 19, with an O/E death ratio of 1.30, and an increased death IRR of 1.47. These results are in agreement with those reported in other studies. For example, Massie et al. found a 41.2% increase in expected deaths and a 1.42 O/E death ratio in KTRs between March 2020 and March 2021 [10].

When investigating probable EM causes, an increased risk of EM was found to be associated with some parameters. COVID-19 introduced a new morbidity and mortality risk globally, and more so in the transplant recipient population. The results of the present study confirm an absolute and relative EM due to COVID-19 infection but not due to other infection etiologies. Furthermore, when looking at deaths in the KTR population during the 10 months (P2C) period that included the second seasonal peak of the COVID-19 pandemic (January 2022), COVID-19 infection accounted for nearly half of deaths from all causes (49%), with an IRR of 1.9, which surpassed expectations.

In line with previous publications, an age-related effect on EM was identified in transplant recipients during the pandemic. Requião-Moura et al. showed that age and time after transplantation were both related to an increased probability of death in KTRs [11]. A systematic review by Roxanne Opsomer et al. demonstrated a consistent correlation between mortality due to COVID-19 and age in SOT recipients, although data supporting transplantation time as an independent risk factor for mortality in severe COVID-19 disease was more disputed [12]. Our analysis showed significantly higher absolute and relative EM by age (between 60 and 70 years-old), and by time since last transplant (over 120 months), and interestingly, considering the infectious risk in the early post-transplant period, we did not find a significant correlation between EM and early time of transplant or younger age. One major explanation is higher mortality due to more age-related comorbidities (cardiovascular and others) in kidney recipient patients with a prolonged active transplant status, considering the long time of exposure of the majority of our transplant cohort samples, which include active grafts that date back as early as 1983, and newly transplanted patients during the study time period were relatively very few.

As expected, we faced limitations. A true case-control mortality model was not possible, since our cohort model had significant overlap between patients from the P1 and P2 transplant recipient cohorts. Also, we should clarify that new kidney transplants during both pre-COVID-19 and the COVID-19 era were included in our sample, as long as the time since the last transplant was at least 6 months. Besides, analysis of excess mortality in a kidney and pancreas transplant recipient subgroup was scarce and inconclusive, and the data was not published. We also lacked data which could allow to stratify the risk of death in the transplant recipient populations for other variables, such as diabetes, smoking, BMI and cardiovascular disease. Conceptually, our statistical approach would not allow for a multivariate analysis.

**FIGURE 2:**
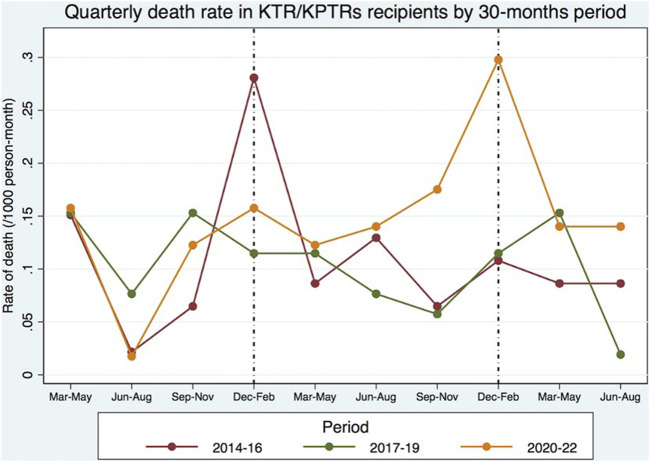
Global mortality in the KTR/KPTR population by 30 month-periods, including in P1 (between 2014 and 2019) and P2 (between March and August 2022) [3].

The comparison of mortality between the cohort of KTRs with the general Portuguese population during the same pandemic time-frame provided interesting results, moreso when searching for different mortality trends during different COVID-19 pandemic waves. To reduce selection bias, we calculated SMR adjusted by age. EM was high in both groups during the first COVID-19 seasonal peak, but the second seasonal peak brought a reduction in mortality rate and EM in the general Portuguese population, together with a much higher rate of primary full-dose SARS-COV-2 vaccination compared to the first peak. Among transplant recipients, the second seasonal peak was associated not only with higher mortality, but also with a several-fold increase in EM (estimated SMR of 9.00), especially in the last 10 months of the study when most (if not all) active KTRs already had full-dose SARS-COV-2 vaccination. The higher mortality is also illustrated in Figure 2, and was partially explained by an approximately 3-fold higher incidence of COVID-19 infection in the general population, mainly during the second seasonal peak. Additional factors may have contributed to the increase in EM observed in the present study, including the shift in social restriction policies that took place in Portugal in 2022 [13, 14]. Figure 2 also shows a seasonal peak in the 2014/2015 winter, probably related to an unusually high EM “from all causes” among the general Portuguese population when compared to the previous winter (global EM of 17%, maximum weekly EM of 36%), largely explained by a high-intensity flu epidemic with unusually low temperatures [15].

CKD is a well-established risk factor for COVID-19-associated mortality [16–21], and questions have arisen about the effectiveness of the COVID-19 vaccine in patients with chronic kidney disease (CKD), end-stage kidney disease (ESKD) on chronic dialysis, and KTR/KPTRs undergoing chronic immunosuppression [22–24].

The seroprevalence of SARS-CoV-2 antibodies has been suggested elsewhere as a measure of vaccine effectiveness [25, 26]. The seroprevalence and decay of SARS-COV-2 antibodies after full-dose vaccination of patients with CKD stages 4 and 5, can be very similar to non-CKD controls [27, 28]. In dialysis patients, the COVID-FRIAT study and other studies reported that the prevalence of antibodies in hemodialysis patients was slightly lower (80–95%), and had an earlier and faster decline compared to the general population [28–31]. Studies on antibody seroprevalence in the KTR/KPTR population showed exceedingly lower values compared to non-transplant counterparts. Sanders *et al.* reported a SARS-CoV-2 seroprevalence after complete vaccination of only 57.7% at day 28% and 49% at 6 months [28]. A Scottish cohort study reported a vaccine effectiveness rate against COVID-19 of only 39% in KTRs who received a full-dose vaccine regimen (i.e., 2 doses of ChAdOx1 nCoV-19 (Vaxzevria) vaccine or 2 doses of mRNA BNT162b2 vaccine) compared to 67–80% in the general UK population [23]. Vaccine effectiveness against hospitalization is also relatively low in this patient population (40%), and the mortality rate due to COVID-19 is 10% versus <0.1% in the general population. Bell et al. compared survival rates in ESKD and KTR patients in two consecutive COVID-19 pandemic waves and showed that survival rates at 28 days after positive SARS-CoV-2 testing in ESKD patients were higher during the second pandemic wave, while in KTRs were nearly overlapping between the first and second waves [23]. It can be hypothesized that this difference is due to the lack of COVID-19 vaccine effectiveness in KTRs, and EM may be an indirect marker of failure of current vaccination strategies among transplant recipients. Interestingly, in other countries, some authors have reported a reduced overall COVID-19 mortality rate among KTRs until 2021 [32, 33], possibly explained by high accessibility to SARS-CoV-2 testing, new treatments, and vaccination, also referring to different timelines and unaccounted confounding factors, such as younger age and less comorbidities, in the study population of the second pandemic wave.

More recently, studies on the serological response in kidney transplant recipients after 3 and 4 doses of a SARS-COV-2 vaccine found higher seroconversion among this population (up to 75%), but Thomson et al. highlight that a significant proportion of transplant recipients still remain seronegative after 3 and 4 doses of SARS-CoV-2 vaccines [34].

Results retrieved from this study emphasize the need for vigilance and a continuous search for alternative therapies to prevent COVID-19 infection and mortality in the solid organt transplant recipient population. Therapies with SARS-COV-2 neutralizing antibodies targeting viral surface proteins in immunocompromised patients seemed promising for SOT recipients, but the rate of appearance of new SARS-CoV-2 strains and the cost and availability of immunoprophylaxis with monoclonal antibodies pose considerable restraints [35]. Recently, the PANAMO study in critically ill patients with COVID-19 infection showed promise using monoclonal antibodies targeting the complement system (C5a), and another study with a neutralizing antibody targeting the receptor binding site for the virus also showed interesting results [36, 37]. Further therapies are also expected to emerge and significantly improve the prognosis of SOT recipients with COVID-19 infection.

## Data Availability

The original contributions presented in the study are included in the article/supplementary material, further inquiries can be directed to the corresponding authors.

## Abbreviations

BMI, Body Mass Index; CHUSA, Centro Hospitalar Universitário de Santo António (Porto, Portugal); CI, Confidence Interval; CKD, Chronic Kidney Disease; DGS, Directorate-General of Health in Portugal (*Direção Geral de Saúde*); EM, Excess Mortality; ESKD, End-Stage Kidney Disease; INE, Statistics Portugal (*Instituto Nacional de Estatística*); IR, Incidence Rate; IRR, Incidence Rate Ratio; KT, Kidney Transplant; KTR, Kidney Transplant Recipient; KPTR, Kidney and Pancreas Transplant Recipient; NA, Not Attributed; O/E, Observed/Expected; RPT, Portuguese Transplantation Registry (*Registo Português de Transplantação*); SER, Portuguese Electronic Health Registry (*Registo de Saúde Eletrónico*); SMR, Standardized Mortality Rate; SOT, Solid Organ Transplant; UK, United Kingdom.
